# Towards the Complete Goat Pan-Genome by Recovering Missing Genomic Segments From the Reference Genome

**DOI:** 10.3389/fgene.2019.01169

**Published:** 2019-11-15

**Authors:** Ran Li, Weiwei Fu, Rui Su, Xiaomeng Tian, Duo Du, Yue Zhao, Zhuqing Zheng, Qiuming Chen, Shan Gao, Yudong Cai, Xihong Wang, Jinquan Li, Yu Jiang

**Affiliations:** ^1^Key Laboratory of Animal Genetics, Breeding and Reproduction of Shaanxi Province, College of Animal Science and Technology, Northwest A&F University, Yangling, China; ^2^College of Animal Science, Inner Mongolia Agricultural University, Hohhot, China

**Keywords:** pan-genome, goats, *de novo* assembly, reference genome, pan-sequences

## Abstract

It is broadly expected that next generation sequencing will ultimately generate a complete genome as is the latest goat reference genome (ARS1), which is considered to be one of the most continuous assemblies in livestock. However, the rich diversity of worldwide goat breeds indicates that a genome from one individual would be insufficient to represent the whole genomic contents of goats. By comparing nine *de novo* assemblies from seven sibling species of domestic goat with ARS1 and using resequencing and transcriptome data from goats for verification, we identified a total of 38.3 Mb sequences that were absent in ARS1. The pan-sequences contain genic fractions with considerable expression. Using the pan-genome (ARS1 together with the pan-sequences) as a reference genome, variation calling efficacy can be appreciably improved. A total of 56,657 spurious SNPs per individual were repressed and 24,414 novel SNPs per individual on average were recovered as a result of better reads mapping quality. The transcriptomic mapping rate was also increased by ∼1.15%. Our study demonstrated that comparing *de novo* assemblies from closely related species is an efficient and reliable strategy for finding missing sequences from the reference genome and could be applicable to other species. Pan-genome can serve as an improved reference genome in animals for a better exploration of the underlying genomic variations and could increase the probability of finding genotype-phenotype associations assessed by a comprehensive variation database containing much more differences between individuals. We have constructed a goat pan-genome web interface for data visualization (http://animal.nwsuaf.edu.cn/panGoat).

## Introduction

A complete and accurate reference genome is fundamental for read alignment and comprehensive discovery of genomic variants. However, there is increasing awareness that a reference genome from a single individual cannot fully represent the genomic diversity of one species since many sequences could be absent in the reference genome. We use the term pan-sequences to describe sequences that are found in other individuals but not in the reference genome. In humans, ∼5 Mb pan-sequences were found to be absent in the reference genome (Li et al., 2010) and more have been identified in subsequent studies (Kidd et al., 2010; Chen et al., 2011; Liu et al., 2014; Liu et al., 2015; Wong et al., 2018). Those pan-sequences could be of high frequency in the populations, demonstrate population stratification, and have biologically important functions (Wong et al., 2018). Therefore, it is necessary to build a pan-genome by uncovering the pan-sequences that are absent from the reference genome to maximally represent the genetic diversity within one species.

As one of the first domesticated livestock species (Zeder, 2008), goats have a broad geographical distribution with excellent adaptations to diverse ecological and agronomic conditions, with 579 local breeds and more than one billion individuals (FAOSTAT, 2016). Thus using the assembly only from a single animal may not be able to adequately explore the rich genetic diversity underlying the diverse breeds and populations of goats. With the rapid advances in sequencing technologies, the generation of high quality *de novo* assemblies is increasingly affordable. The goat reference assembly (ARS1) is the first genome in livestock generated using long-read sequencing technology which has comparable continuity with the current human genome and is thus considered a ‘golden’ genome (Bickhart et al., 2017; Worley, 2017). Meanwhile, genome assemblies for many Caprini species are publicly available including those of *Capra aegagrus* (bezoar) (Dong et al., 2015), *Ovis aries* (domestic sheep) (Jiang et al., 2014), *Ovis musimon* (mouflon) (Naval-Sanchez et al., 2018), *Ovis ammon* (argali) (Yang et al., 2017), and *Capra sibirica* (Siberian ibex).

The construction of pan-genome relies on a number of *de novo* representative assemblies (Golicz et al., 2016), which is hampered by the high cost of generating *de novo* assemblies. Fortunately, many *de novo* assemblies from Caprini species have been generated that are all closely related with goats. Notably, a recent study found that many of the sequences missing from the human reference genome are ancestral sequences (Wong et al., 2018), indicating that the missing sequences could still persist in the closely related species. We, therefore, applied a strategy to explore the missing goat sequences from the reference genome by utilizing available Caprini *de novo* assemblies. The unaligned sequences from each Caprini assembly as compared with the goat reference genome were collected, and their presence in goats was further validated by goat whole-genome resequencing and transcriptomic data. The identified sequences will not only complete the goat reference genome but also enable a more comprehensive repertoire of goat genomic variations to be compiled.

## Results

### Genomic Divergence Among the Caprini Genomes

**Figure 1:**
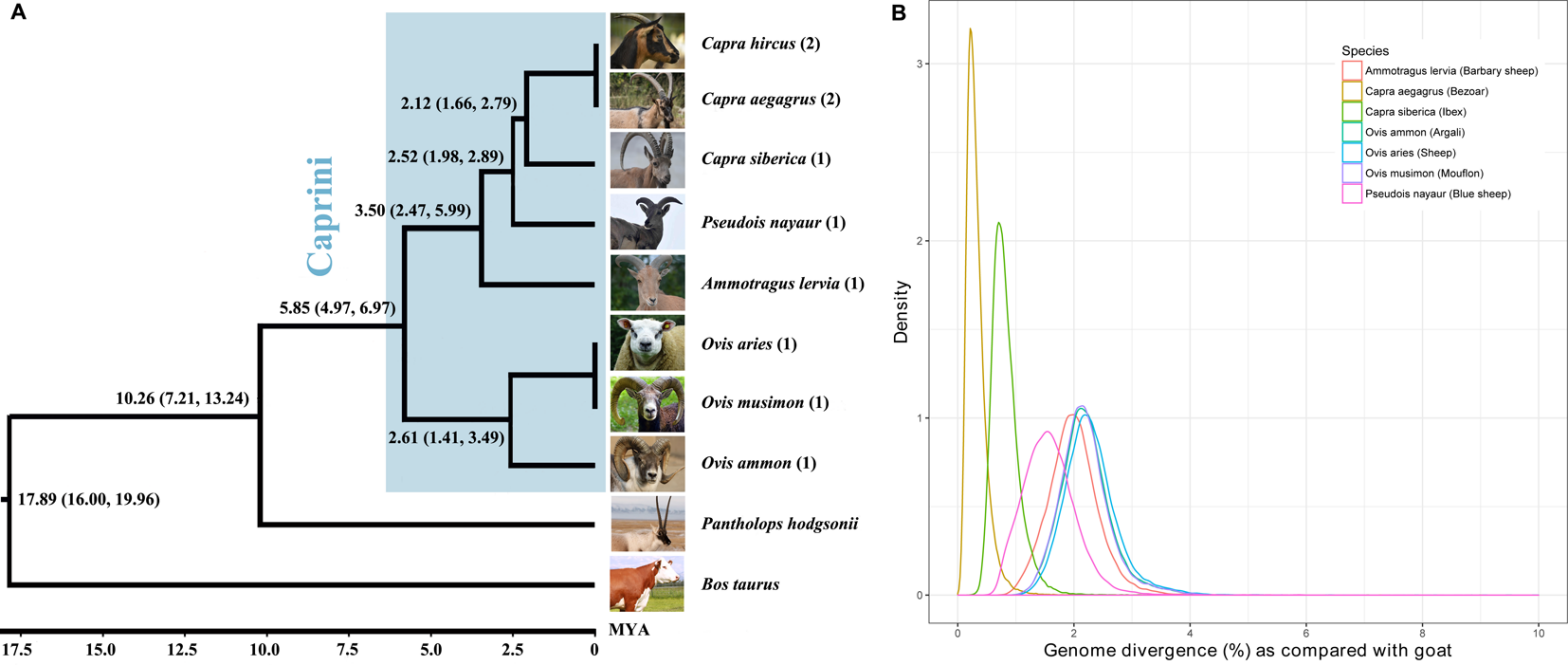
Phylogenetic relationship of Caprini species **(A)** and their genomic divergence **(B)**. Each of the representative genomes of other Caprini species was compared with the goat reference genome to estimate the genomic divergence.

By comparing each genome to the goat reference genome (ARS1) using LAST (Kiełbasa et al., 2011), the genomic divergence between each assembly with the goat was calculated by counting the percentage of nucleotide divergence sites within the 1:1 alignment. The genomic divergence was very low among all of the Caprini species, ranging from 0.21% (bezoar versus goat) to 2.18% (sheep versus goat) (**Figure 1B**). The genomic divergence between the Caprini species with goat correlates well with their evolutionary relationship (**Figure 1A**).

By comparing other Caprini genomes with ARS1, we can extract the ‘ancestral’ sequences that are absent from ARS1. It should be noted that the genomic divergence rates among Caprini species were very small, as described above, indicating that short reads mapping from goat resequencing data to these sequences would be feasible (Li and Durbin, 2010). Thus, based on whole genome resequencing data of goats, we can verify their existence in goats and correct the divergence sites to corresponding goat nucleotides to represent true goat sequences.

### Recovery of Sequences Missing in the Present Goat Reference Genome

We first extracted the unaligned sequences from each Caprini assembly compared with ARS1, verified their existence in goats, and finally the sequences belonging to goats based on whole genome resequencing data from goats. Translocation and inversion events are excluded from the dataset. The goat ARS1 is the most continuous genome for animal species and thus is used as the reference for comparison with nine other assemblies of Caprini species. As described in the methods section, each of the assemblies was iteratively compared with ARS1 to collect unaligned sequences. In this way, we obtained a total of 153.8 Mb of additional sequences with a minimum length of 400 bp.

**Figure 2:**
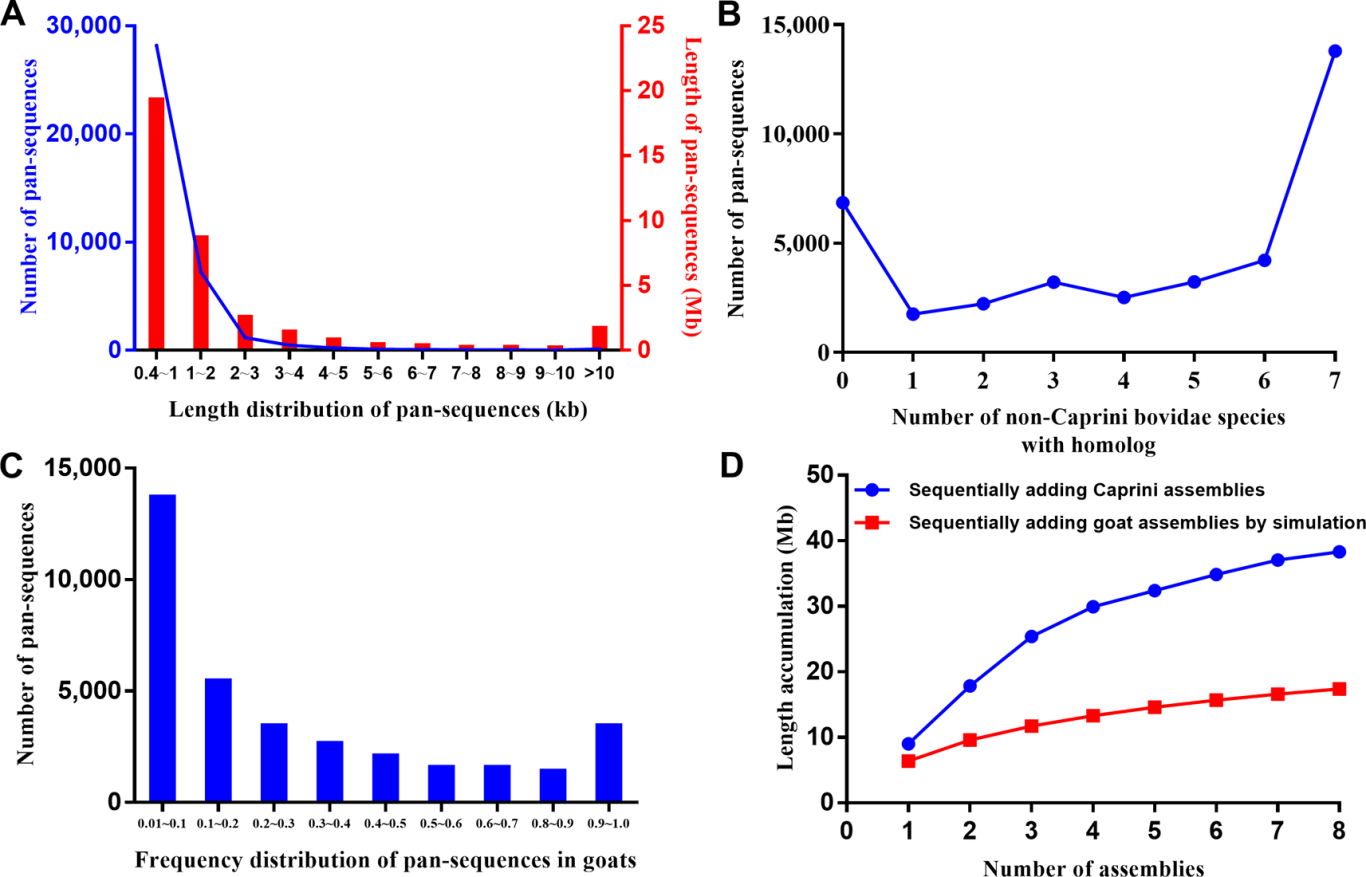
Characteristic of pan-sequences. **(A)** Length distribution of pan-sequences. **(B)** Homolog identification of pan-sequences within seven non-Caprini bovid species. **(C)** Frequency distribution of pan-sequences in domestic goats. **(D)** The cumulative size of pan-sequences by sequentially adding *de novo* assemblies of eight Caprini species (blue line) as compared with simulated sequence length by adding goat individuals (red line). The simulated sequence length was calculated using the formula as described in methods.

These additional sequences could either represent true goat sequences missing in ARS1 or simply fixed genomic differences between goat and other species. To validate the presence of each sequence in goats, the primary pan-genome (ARS1 plus additional sequences) was used as the reference for reads mapping of all the resequencing data of 107 goats and 20 bezoars. Based on the mapping information, the normalized haploid read depth (NRD) of each sequence was calculated. Only those with NRD ≥0.4 in at least two samples of goats were considered as authentic goat sequences. Since those goat pan-sequences were inferred from other Caprini species, they still contain divergent sites. To construct a true goat pan-genome, we replaced all the divergent sites with corresponding goat nucleotide bases on the whole genome resequencing data. In this way, a total of 37,840 sequences adding up to 38.3 Mb were found to be present with the frequency of at least two out of 107 individuals and were thus defined as goat pan-sequences. The pan-sequences had an average length of 1,013 bp ranging from 400 bp to 33 kb (Figure 2A). We further aligned those sequences to seven non-Caprini bovidae species (*Aepyceros melampus*, *Connochaetes taurinus*, *Litocranius walleri*, *Cephalophus harveyi*, *Oryx gazelle*, *Bos taurus*, and *Pantholops hodgsonii*) to search for any matches or homologs and found that 82.9% (31,357/37,840) of the sequences could be aligned (Figure 2B). Approximately 40% of the pan-sequences had a frequency of 0.01–0.1 within the studied goat populations whereas only ∼1% were widely present with a frequency of >0.9 (Figure 2C). Thus, we constructed a goat pan-genome composed of the base assembly (ARS1, referred to as pan-base) and the 38.3 Mb pan-sequences (**Supplementary Table S1**).

Furthermore, the results depicted that using *de novo* assemblies from closely related species could recover more pan-sequences than using the same number of goat *de novo* assemblies (**Figure 2**). Thus, the inter-species comparison is an efficient method for the recovery of pan-sequences and will likely greatly reduce the required number of *de novo* assemblies.

### Characterization of Pan-Sequences

**Figure 3:**
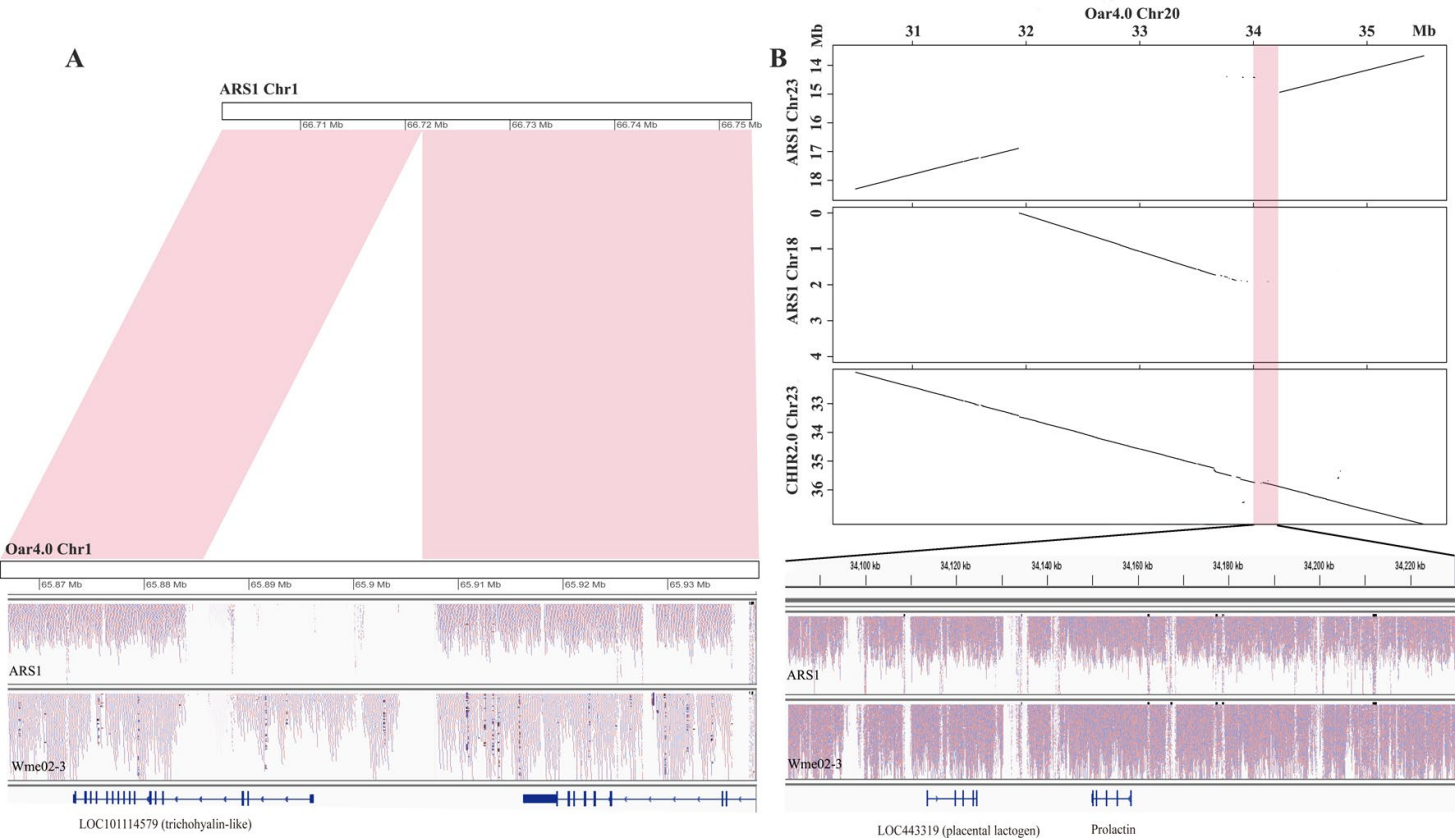
The source of pan-sequences. **(A)** An example of pan-sequences resulting from insertions. A region of 18.8 kb was found to be present in goat by comparing Oar4.0 with ARS1 which was supported by reads mapping information. **(B)** An example of pan-sequences resulting from assembly errors in ARS1. The dot plots showed a region of 148 kb identified from chr20 of Oar4.0 that was missing in chr23 of ARS1. The presence of this region was supported by synteny with chr23 of CHIR2.0 and by the reads mapping information.

To assess whether the pan-sequences were presence/absence variants relative to ARS1, we first examined the NRD values of each pan-sequences in the sequenced ARS1 individual. A total of 25.97 Mb was found to be absent from the sequenced individual (NRD ≤ 0.2), indicating that most of the pan-sequences were likely presence/absence variants. For example, an 18.8 kb insertion was found on chr1, which partially covered the genic region of a trichohyalin-like gene (**Figure 3A**). The presence of the insertion could be further supported by the resequencing data. We also found that 9.66 Mb sequences were actually present in the sequenced individual of ARS1 (NRD ≥0.4) in spite of their absence in reference assembly, which was probably due to the limitation of sequencing or assembling method. In particular, a region of ∼2 Mb sequences that were supposed to be on chr23 was misplaced on chr18 accompanied by the absence of a 148 kb adjacent region (chr23: 34,082 kb–34,230 kb) (**Figure 3B**). This missing region covered prolactin (PRL) and placental lactogen (LOC443319) genes, which are crucial regulators of lactation (Bolefeysot et al., 2011). In addition, we identified 1.18 Mb pan-sequences that were most likely divergent alleles. Also, we aligned the pan-sequences to ARS1 with their flanking sequences and found that 66.8% of the sequences could be successfully positioned on ARS1. The anchoring information not only validated the presence/absence variants but also enabled the visualization of the pan-sequences on ARS1 with the pan-genome website as described below.

We next explored the genes contained in the pan-sequences. A total of 2,564 sequences were annotated to the coding region 1,206 genes with the largest number of sequences (227) belonging to LOC102334977, an uncharacterized protein followed by LOC108638286 (156), a keratin-associated protein. Some of the pan-sequences and their descriptions are shown in **Table 1**. Using the RNA-seq data from nine goat tissues, 305 sequences containing exonic regions from 216 genes showed appreciable expression (FPKM > 1 in at least one sample). Furthermore, additional 1,248 sequences demonstrated expression but lacked appropriate annotation, which could be due to the limitation of annotation of short sequences. The pan-sequences were further explored at the protein level by aligning them to all available RefSeq proteins from goat, cattle, and sheep using BLASTX (e-value < 1e-5) (**Supplementary Figure S1**). Among the RefSeq gene mapping results, the most abundant of the hits were members of KRAB domain containing protein (20.1%), which belong to a large family of zinc finger proteins,(Collins et al., 2001). Further protein categories were composed of highly variable families including glypican and EF-hand calcium-binding domain-containing protein.

**Table 1:** Examples of pan-sequences and their annotated genes.

Pan-sequences ID	Number of samples with NRD ≥0.4	Length (bp)	Identity (Coverage) with ARS1^a^	Paralog copies in goat pan-genome^b^	Annotated genes	Gene description
3_213322000_213345586-Ovis.aries	107	23586	No hit	0	LOC101119130	TRIO and F-actin-binding protein
5_12421945_12429000-Ovis.aries	11	7055	76 (31)	1	LOC101112563	Intercellular adhesion molecule 1-like
6_115990993_115994660-Ovis.aries	107	3667	70 (37)	1	FGFR3	Fibroblast growth factor receptor 3
8_73765000_73783000-Ovis.aries	107	18000	84 (11)	0	LRP11	Low density lipoprotein receptor-related protein 11
12_71555499_71581526-Capra.hircus	20	26027	75 (17)	6	MRP4	Multidrug Resistance-Associated Protein 4
18_19266000_19273036-Ovis.aries	102	7036	77 (38)	4	LOC101102268	myeloid-associated differentiation marker-like
20_25417856_25432000-Ovis.aries	7	14144	84 (16)	1	LOC101109220	SLA class II histocompatibility antigen, DQ haplotype D alpha chain-like
20_34142966_34162204-Ovis.aries	107	19238	77 (19)	4	PRL	Prolactin
AJPT02077673.1_24914_38585-Capra.hircus	15	13671	82 (13)	1	DQA1	SLA class II histocompatibility antigen, class II, DQ alpha 1
AJPT02103288.1_12703_24009-Capra.hircus	66	11306	78 (22)	2	GBP7	Guanylate binding protein 7

^a^The identity (coverage) of each pan-sequences were determined using BLASTN. ^b^The paralog copies were determined using tblastx (evalue<1e-20).

Based on the distribution (presence/absence variants) of each pan-sequence, we performed genetic structural analysis by clustering all the individuals into groups without prior information of individual origins (**Supplementary Figure S2**). The samples generally clustered by geographical distributions. The first group is composed of East Asia and a small number of West Asia samples whereas the other group included African, European, remaining West Asia, and the bezoar samples. The distribution pattern of pan-sequences indicated that they were preferentially retained in diverse populations and might confer benefits that favor local adaptations.

### Improved Genomic and Transcriptomic Mapping Efficacy Using the Pan-Genome

**Figure 4:**
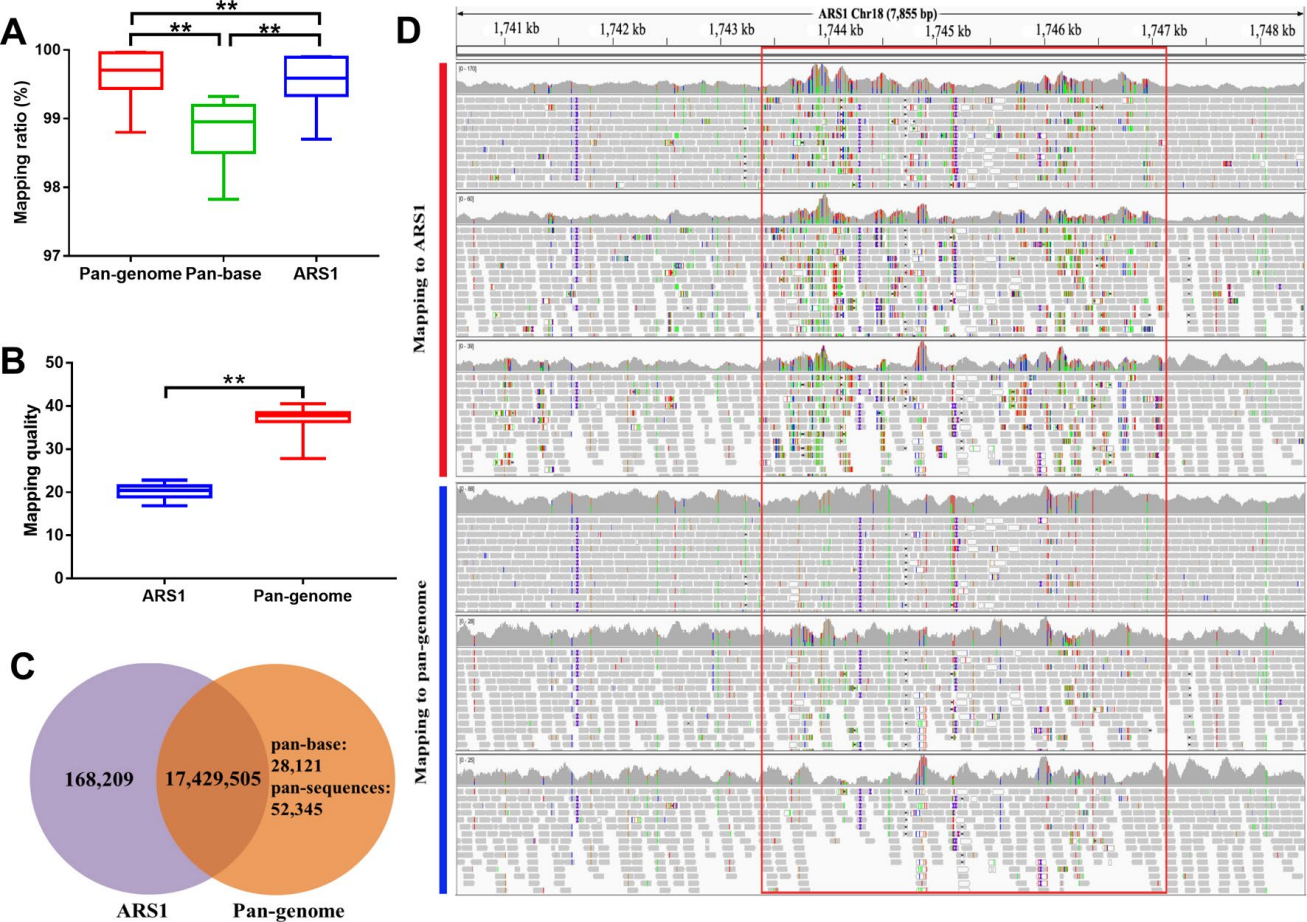
Improvement of reads mapping for resequencing data using pan-genome versus ARS1. **(A)** Comparison of mapping ratio of resequencing data using pan-genome versus ARS1. **(B)** The mapping quality of reads from pan-sequences as compared with their original mapping quality on ARS1. **(C)** The number of identified SNPs for the 10 goat samples using pan-genome versus ARS1. **(D)** The reads mapping quality was improved within the red rectangle accompanied by repression of false SNPs removal of the low-quality mapped reads. Pan-base specifically refers to the ARS1 proportion in the pan-genome when using the pan-genome as the reference for mapping whereas ARS1 refers to using the ARS1 as the reference for mapping. T-test was used for the comparison. ** *P* < 0.01.

Compared with ARS1, the genomic mapping ratio of resequencing data was increased by 0.04%–0.15% in the pan-genome. We further noticed that the mapping ratio of the pan-base was reduced by ∼0.75% indicating that many reads have been adjusted to their rightful positions in the pan-sequences (**Figure 4**). The reads that were mapped on pan-sequences demonstrated higher mapping quality as compared with their original mapping positions in ARS1 (**Figure 4**). Therefore, the pan-sequences will not only help to increase the mapping rate but also allow better positioning for the mapped reads. In addition, the number of spurious SNPs is expected to be greatly reduced whereas more novel and confident SNPs will be recovered. To evaluate the degree of improvement in SNP calling, we randomly selected 22 goat samples and compared the difference in the resulting SNPs using the pan-genome compared with ARS1. A total of 168,209 spurious SNPs were repressed as a result of better reads mapping quality (**Figures 4C, D**). We also found three paralogs of this region with coverage of 67–90% and identity of 89–92% using BLASTN. The spurious SNPs were most likely to reside in not conservative regions containing paralogs or repeats which can result in misalignments of short reads. Furthermore, the missing of the real sequences that the short-reads belong to will increase the possibility of misalignment accompanied by occurrence of false SNPs. Meanwhile, 80,466 novel SNPs were identified based on the pan-genome including 52,345 SNPs from the pan-sequences.

**Figure 5:**
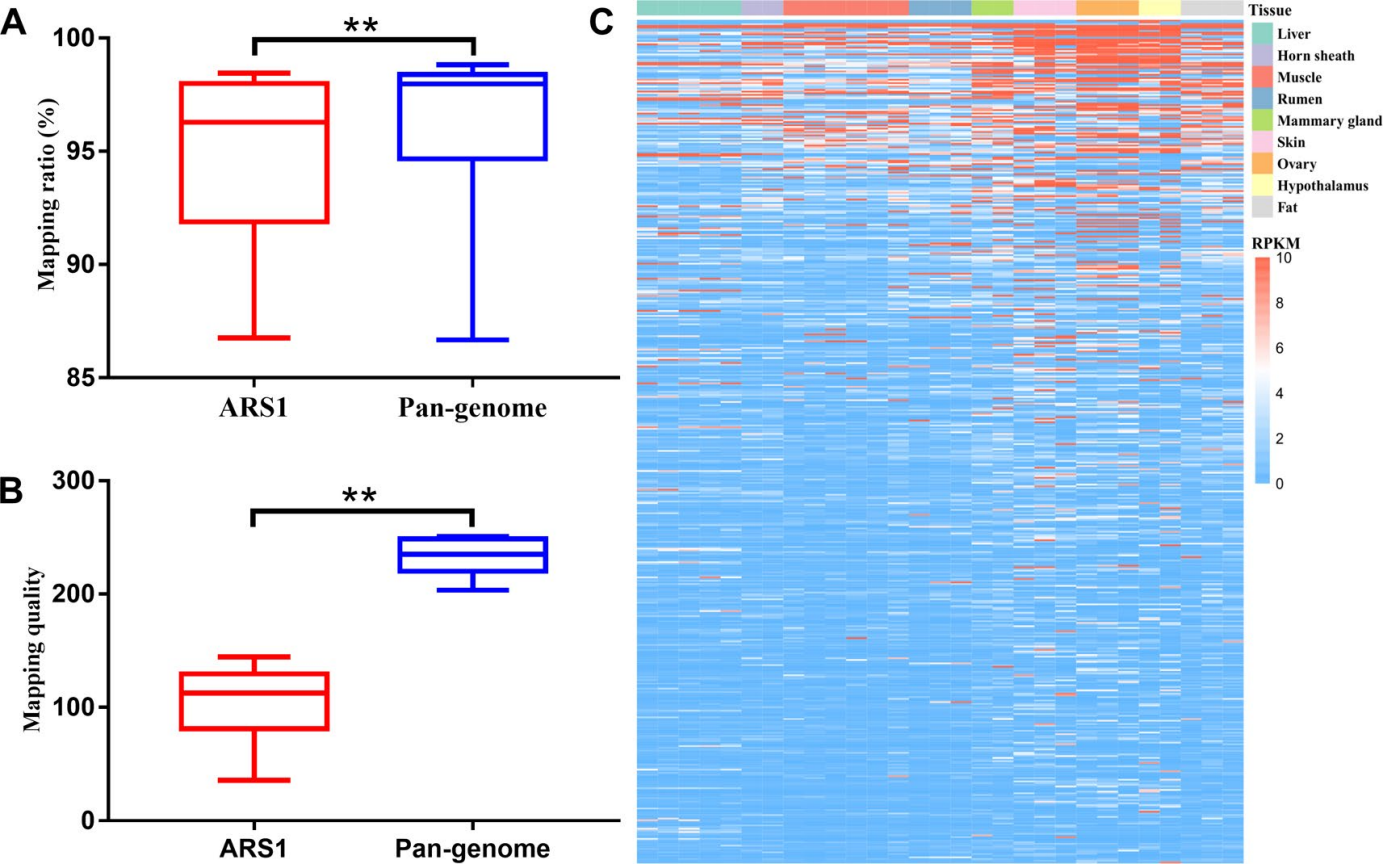
Improvement of reads mapping for transcriptomic data using pan-genome versus ARS1. **(A)** Comparison of mapping ratio of resequencing data using pan-genome versus ARS1. **(B)** The mapping quality of reads from pan-sequences as compared with their original mapping quality on ARS1. **(C)** The expression of pan-sequences across nine tissues. T-test was used for the comparison ** *P* < 0.01.

We further evaluated the mapping ratio of 20 RNA-seq datasets using the pan-genome compared with ARS1. The transcriptomic mapping rate was increased by ∼1.15% using the pan-genome compared with ARS1 (**Figure 5A**). Furthermore, the mapping quality of reads mapped to pan-sequences was also greatly improved compared with their originally mapped positions on ARS1 (**Figure 5B**). Thus 2,564 sequences were shown to be expressed with FPKM ≥1 in at least one sample (**Figure 5C**).

### Construction of Goat Pan-Genome Website

**Figure 6:**
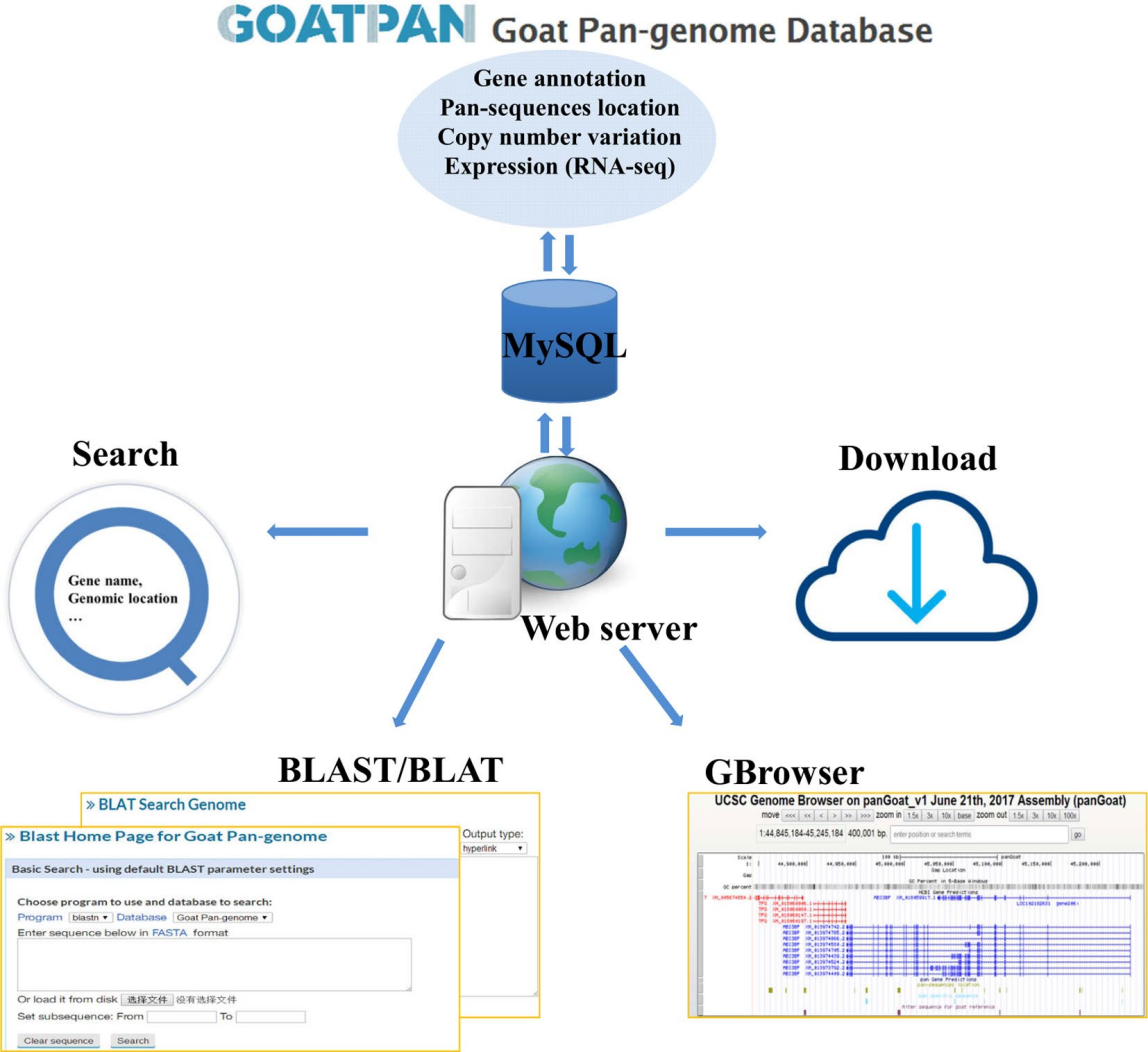
Overview of goat pan-genome database features.

To make our pan-genome available for public use, we have constructed a user-friendly web interface for browsing and downloading the goat pan-genome (GOATPAN) (**Figure 6**). Users can input the gene symbol or alias of interest or genomic coordinate to view pan-sequence information (gene annotation, genome location, and haploid copy number for each individual). Users can also retrieve the haploid copy number variations, gene annotation, and expression level information of the pan-sequences as well as the whole pan-genome. The sequence data of the pan-genome can be freely downloaded. Our browser will be updated regularly by incorporating resequencing data from more individuals to support various needs from the scientific community. The GOATPAN will accelerate genomic studies and molecular breeding in goats by serving as a better and more comprehensive reference.

## Discussion

In this study, we used multiple Caprini *de novo* assemblies to identify the missing goat sequences from the reference genome. The most ideal strategy is to use multiple *de novo* assemblies representing diverse goat populations as applied for humans in a recent study (Wong et al., 2018). However, generating multiple high-quality assemblies remains a big challenge due to the high cost and the complexity of mammalian genomes. Therefore, we provided an alternative strategy by using *de novo* assemblies from closely related species. The Caprini species that we used have high genomic similarity and are highly conserved, allowing the use of a greatly reduced number of individuals to build a pan-genome that otherwise could have required hundreds of goat *de novo* assemblies. A further validation using whole genome resequencing data from goats confirms the authenticity of these recovered sequences, thereby suggesting that this strategy is feasible and potentially applicable to other species.

Despite the considerable number pan-sequnces we recovered, the goat pan-genome size would be much larger than we expected. Recent advancements in the human pan-genome revealed ∼10% of genomic sequences missing from the reference genome (Sherman et al., 2019). Our stargety take advange of genomic information from related species to recover missing sequences of one organism. This approach, however, has its limitations. First, the *de novo* assemblies were from eight Caprini species sharing a common ancestor 5.85 million years ago. The divergence times between these species indicate that many sequences could only be found using goat *de novo* assemblies. Second, our approach would underestimate the contribution of paralogs, segmental duplications, and TEs to the pan-genome. We used an identity cutoff of 95% to determine pan-sequences whereas the young paralogs and segmental duplications generated within millions of years would still share high identities (>95%) (Bailey et al., 2003; Alkan et al., 2009). In addition, TEs could expand greatly over short evolutionary periods due to their replicative nature and continuous accumulation (Platt et al., 2018). Therefore, in order to build the complete goat pan-genome, a better strategy still need to rely on more high-quality assemblies representing worldwide goat populations. We anticipate that this would become feasible in the following years with the advances in sequencing technologies and rapid reduction in sequencing cost, especially from long-read sequencing.

We recovered a considerable number of missing sequences in the reference assembly (ARS1), most of which stem from presence/absence variants. Notably, we found that some sequences containing essential genes were missing from ARS1, despite their presence in the sequenced ARS1 individual. This finding suggests that the current long-reads sequencing technologies or the assembling methods still have limitations that warrant further improvements. The significance of the recovery of missing sequences from the human genome has been well acknowledged especially in recent years (Kehr et al., 2017; Ameur et al., 2018). However, the extent and size of missing sequences from other mammalian species are largely ignored (Peona et al., 2018). Considering the much lower quality of the reference genomes for other species compared with human, this kind of work is highly demanding for livestock and other important species that require a high-quality genome for breed selection and improvement.

The identified pan-sequences will enable improved genomic and transcriptomic studies, which have also been confirmed in humans (Ameur et al., 2018). One of the important applications is improved SNP calling. SNPs are considered to be among the most important genomic variations and are essential in genomic studies (such as genome-wide association studies, maker assisted selection, and population diversity analysis). SNP calling relies on read mapping, followed by processing of the mapped reads, variant calling, and variant filtering (Nielsen et al., 2011). Most studies to date have mainly relied on a single reference genome to call SNPs between multiple individuals. Since a considerable number of reads have been adjusted to better positions in the pan-sequences, the overall number of identified SNPs can be increased by considering the pan-sequences. In addition, the reads mapping quality in the reference proportion will be improved, thereby reducing the number of mistaken SNPs. Furthermore, the identification of additional SNPs, which would not have been possible using a single reference alone, could be potentially associated with economic and local adaptation traits (Kehr et al., 2017) and could serve as genetic markers for animal breeding and selection.

## Conclusions

In this study, we recovered abundant goat pan-sequences that are absent from the reference genome, which will avoid bias due to missing sequences and ensure full representation of genomic diversity within one species. We show that using *de novo* assemblies from closely related species is a feasible strategy for pan-genome construction when multiple *de novo* assemblies are not available.

## Methods

### Phylogenetic Relationship of Caprini Species

We have retrieved 10 publicly available assemblies in NCBI including domestic goat representative (*Capra hircus*, ARS1) (Bickhart et al., 2017) and alternate (*Capra hircus*, CHIR_2.0) (Dong et al., 2013) assemblies, bezoar representative (*Capra aegagrus*, CapAeg_1.0) (Dong et al., 2015) and alternate (*Capra aegagrus*, Caeg1) assemblies, sheep representative assembly (*Ovis aries*, Oar4.0) (Jiang et al., 2014), mouflon assembly (*Ovis musimon*, Oori1), argali assembly (*Ovis ammon*, Argali1.0) (Yang et al., 2017), Siberian ibex (*Capra sibirica*, CSI1.0), barbary sheep (*Ammotragus lervia*, ALER1.0), and blue sheep (*Pseudois nayaur*, ASM318257v1) (**Supplementary Table S2**). The one-to-one orthologous gene pairs between four sheep and humans, sheep and cattle were downloaded from the ENSEMBL BIOMART database (release 90). The intersection of these two pairs was retained as the one-to-one orthologous gene list in sheep, from which we extracted the four-fold degenerate sites using the sheep genome annotation file from NCBI. Then the four fold degenerate sites of the goat genome (ARS1), cattle genome (BTAU5.0.1), and chiru genome (PHO1.0) were extracted using an in-house script. The corresponding four degenerate sites for *Capra aegagrus*, *Capra sibirica*, *Pseudois nayaur*, *Ammotragus lervia*, *Ovis aries*, *Ovis ammon*, and *Ovis musimon*, were extracted based on their mapping information to the pan-genome. The resulting 689,331 four-fold degenerate sites were used to reconstruct the phylogenetic tree using RAxML using model GTRGAMMA (Stamatakis, 2014). The MCMCtree Bayesian program in the PAML v4.4 package (Yang, 2007) was used to estimate the divergence times within tribe Caprini using a relaxed molecular clock. Multiple calibration-times from fossil records were used in the divergence time estimation including those from bovid (Solounias et al., 1995) and Caprini (Van Dam et al., 2001; Mead and Taylor, 2005; Bibi et al., 2012) species and more details were provided in **Supplementary Table S3**.

### Genomic Divergence Estimation

The genomic divergence and synteny among Caprini genomes were determined by the alignment generated using LAST program (-m100 -E0.05) (Kiełbasa et al., 2011). Each of the representative assemblies for sheep (Oar4.0), argali (Argali1.0), mouflon (Oori1), bezoar (CapAeg_1.0), ibex (CSI1.0), blue sheep (ASM318257v1), and barbary sheep (ALER1.0) were compared with the goat genome (ARS1) to generate the 1:1 alignment. The resulting 1:1 alignment was 2.41 Gb, 2.40 Gb, 2.35 Gb, 2.43 Gb, 2.54 Gb, 2.34 Gb, and 2.41 Gb for sheep, argali, mouflon, bezoar, ibex, blue sheep, and barbary sheep respectively. The genomic divergence between each of the Caprini species with the goat was then calculated by counting the percentage of total nucleotide divergence sites divided by the total length of 1:1 alignment (Yang et al., 2017).

### Identification of Missing Sequences From the Goat Reference Genome

The goat representative assembly (ARS1) is one of the most contiguous genomes in livestock and was thus used as the guided reference genome. The unaligned sequences from Oar 4.0 were first extracted (≥400 bp) and added to goat ARS1 to form the primary pan-genome. Then each of the other assemblies was iteratively aligned to the primary pan-genome as we describe below. The unaligned sequences from each assembly were compared with each other to remove redundancy using CD-hit (-c 0.95 -aS 0.8 -d 0 -sf 1 -M 10000) (Fu et al., 2012), resulting in additional sequences for each assembly. For comparison, each assembly was shredded into 1 kb fragments and aligned to ARS1 using BLASR to extract low similarity sequences with identity below 90% or those without a hit (Chaisson and Tesler, 2012). Then BLASTN (-dust no) (Camacho et al., 2009) was applied to filter those with identities below 95%. In this way, we obtained a goat primary pan-genome including ARS1 and the additional sequences.

To Validate the Authenticity of the Additional Sequences, We Aligned All of Them to the *De Novo* Assemblies of Seven Bovid Species (*Aepyceros Melampus*, *Connochaetes Taurinus*, *Litocranius Walleri*, *Cephalophus Harveyi*, *Oryx Gazelle*, *Bos Taurus*, and *Pantholops Hodgsonii*) to Search for Any Matches or Homologs (≥80% Identity and ≥50% Coverage) Using BLASR (–Bestn 1 –Affineextend 0 –Affineopen 8 –Sdptuplesize 13 –Nosplitsubreads) (Chaisson and Tesler, 2012).

### Correcting Divergence Sites in Pan-Sequences to Represent Goat Sequences

Since the missing sequences from ARS1 were identified from other Caprini species, they still contain divergence sites that need to be corrected to the corresponding goat nucleotide bases. A total of 40 whole genome sequence data representing diverse goat populations were used for correction using Pilon (–diploid –fix all,breaks –mindepth 1) (Walker et al., 2014). For each resequencing data, the reads mapped to pan-sequences was first extracted using Samtools v1.3 and then realigned to pan-sequences using BWA-mem with default parameters to generate the alignment BAM file. With the generated BAM file as input, the pan-sequences were corrected using Pilon. Then the corrected pan-sequences was subjected to a new round of correct by another whole genome sequencing data using the same procedure as described above.

### Gene Annotation

Repeat sequences were identified using a combination of RepeatMasker v4.0.5 (Tarailo‐Graovac and Chen, 2009) with RepBase-20170127 and RepeatModeler V1.0.8 (http://www.repeatmasker.org) and were then masked before gene finding. We then used homology-based and *de novo* prediction to annotate protein-coding genes. For homology-based prediction, protein sequences from five different species (*Bos taurus*, *Ovis aries*, *Sus scrofa*, *Equus caballus*, and *Homo sapiens*) were mapped onto the repeat-masked assembly using TblastN with an E-value cutoff of 1e-5. Aligned sequences, as well as corresponding query proteins were then filtered and passed to GeneWise (Birney et al., 2004) to search for accurately spliced alignments. For *de novo* prediction, we randomly selected 1,500 full-length genes from the results of homology-based prediction to train the model parameters for Augustus v3.2.1 (Stanke et al., 2008) and geneid v1.4.4 (Blanco et al., 2007). GenScan (Burge and Karlin, 1998), Augustus v3.2.1 (Stanke et al., 2008), and geneid v1.4.4 (Blanco et al., 2007) were then used to predict genes using default parameters as described in our previous study (Yang et al., 2017).

### Mapping of Resequencing and Transcriptomic Data to Goat Primary Pan-Genome

We collected whole genome resequencing data for 107 domestic goats (*Capra hircus*) and 23 wild goats (*Capra aegagrus*) for validation and population structure analysis of the pan-sequences (**Supplementary Table S4**). We also downloaded 29 RNA-seq data from nine goat tissues including some unpublished data. These data were from mammary gland, liver, hypothalamus, horn sheath, muscle, rumen, ovary, skin, and testis (**Supplementary Table S5**) and were used to determine the expression level of the pan-sequences.

For the Resequencing Data, Clean Reads From Each Sample Were Mapped to Our Goat Primary Pan-Genome Using BWA-Mem (Li and Durbin, 2009) With Default Parameters. the Haploid Read Depth for Each Pan-Sequence Was Normalized Towards the Genome-Wide Median Read Depth Using Cnvcaller Program With Window Size of 200 Bp and Step Size of 100 Bp to Generate the Normalized Haploid Read Depth (NRD) (Wang et al., 2017). a Pan-Sequence Was Considered to Be Present in Goat (Defined as Pan-Sequences) With NRD of ≥ 0.4 in At Least Two Individuals.

The RNA-seq data were first mapped to the pan-genome using HISAT2 (Kim et al., 2015) and transcript assembly and quantification were performed using Stringtie (Pertea et al., 2015) with default parameters. Then, Ballgown was used to calculate the expression level of each pan-sequence (Pertea et al,. 2016).

### Estimating the Discovered Size of Pan-Sequences Using Goat *De Novo* Assemblies

The estimation was performed using the strategy described by Li et al. (2010) with minor modifications. Briefly, the individual specific sequences were ∼6.4 Mb in length when comparing CHIR2.0 (a Yunnan black goat from China) with ARS1 (a San Clemente goat from the US), which was used as the upper bound between two individuals due to the deep divergence between Western and Eastern goats. Using a transformed Watterson’s θ which was originally an estimator of SNP divergence, the total size of individual-specific sequences was estimated using the following formula:

$$K=\theta \times L\times a,a=1+\frac{1}{2}+\frac{1}{3}\ldots +\frac{1}{n}$$

where n is the effective population size (Ne) of domestic goat, L is the size of a single goat genome, and θ is the averaged individual specific sequence rate. Instead of using the overall population size for n, we used Ne, since it is more appropriate to represent the actual breeding population and the genetic diversity. The range of (θ×L) was roughly estimated to be equal to the divergence between CHIR2.0 and ARS1(∼6.4 Mb). The effective population size of goats was estimated to be 11,574 based on our study (unpublished), resulting in an estimated a of 9.9. Subsequently, the size of pan sequences can be estimated with the increase in the *denovo* assemblies

### Annotation of Pan-Sequences

The annotation of pan-sequences stemming from publicly available genomes was based on their corresponding annotation files (gff3 files). For the other pan-sequences, we used a homology-based method to annotate protein-coding genes. Protein sequences from five different species (cattle, horse, human, sheep, pig) were mapped onto the repeat-masked sequences using TblastN (evalue < 1e-5); the aligned sequences as well as the corresponding query proteins were then filtered and passed to GeneWise (Birney et al., 2004) to search for accurately spliced alignments.

## SNP Calling

To verify whether using the pan-genome as a reference could improve SNP calling, we randomly selected sequence reads from ten goat samples (ranging from 10 to 30× coverage) and mapped the clean reads of each sample to the pan-genome and ARS1. Duplicate reads were removed using Picard Tools. Then, the Genome Analysis Toolkit (GATK, version 3.6) was used to detect SNPs. The following criteria were applied to all SNPs: (1) mean sequencing depth (for all included individuals) > 1/3× and <3×; (2) Variant confidence/quality by depth (QD) > 2; (3) RMS mapping quality (MQ) > 20.0.

## Author Contributions

YJ designed the study and supervised the analysis. RS and JL collected the samples. RL, WF, RS, XT, DD, YZ, ZZ, QC, SG, and YC performed the experiments and analyzed the data. RL wrote the manuscript. YJ and XW revised the paper.

## Conflict of Interest

The authors declare that the research was conducted in the absence of any commercial or financial relationships that could be construed as a potential conflict of interest.

## Supplementary Material

Supplementary Figure S1 The number of alignment hits in pan-sequences of each protein family. RefSeq proteins were collected and aligned against the pan-sequences using BLASTX (e-value<1e-5). Only the best hit was retained on each novel sequence location.

Supplementary figure S2 Clustering of the 107 domestic goats and 20 bezoars using presence/absence information of pan-sequences. The presence of each pan-sequence (rows) was shown in red while the absence of each pan-sequence was shown in blue.
