# Supplementary figures and images for: Towards the Complete Goat Pan-Genome by Recovering Missing Genomic Segments From the Reference Genome
Source: Front Genet. 2019 Nov 15;10:1169. doi: 10.3389/fgene.2019.01169 (PMC6874019; doi:10.3389/fgene.2019.01169)

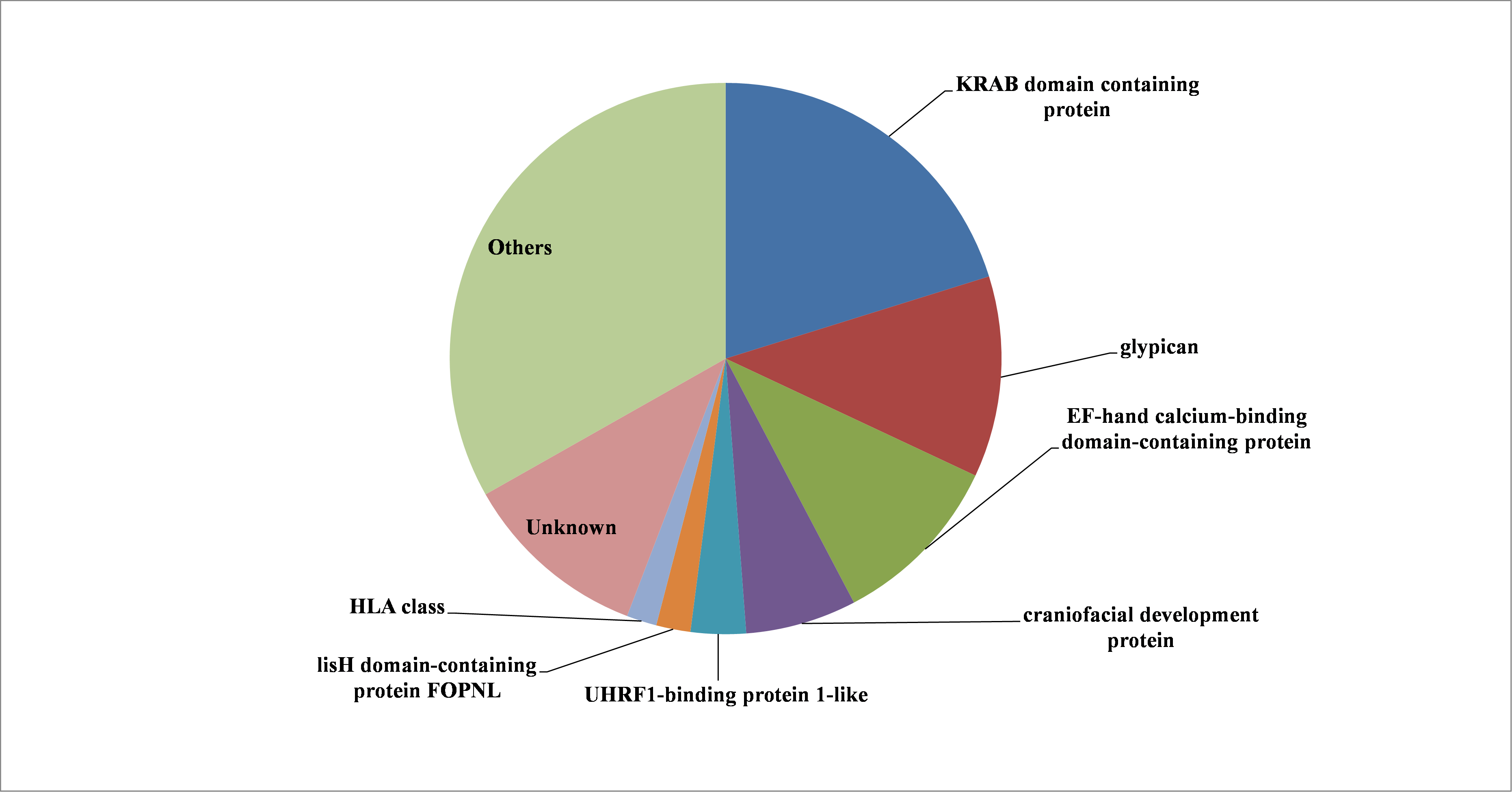

Supplement: Supplementary Figure S1 — The number of alignment hits in pan-sequences of each protein family. RefSeq proteins were collected and aligned against the pan-sequences using BLASTX (e-value<1e-5). Only the best hit was retained on each novel sequence location. [file Image_1.png]

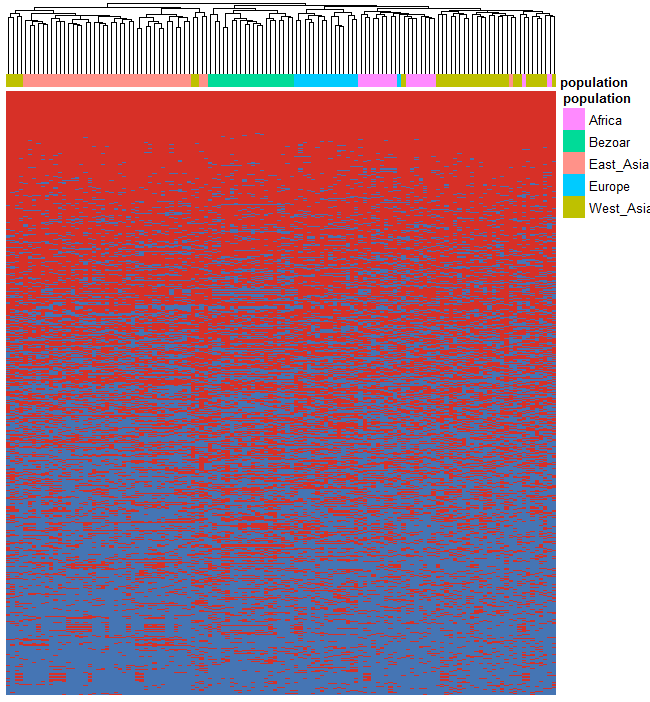

Supplement: Supplementary figure S2 — Clustering of the 107 domestic goats and 20 bezoars using presence/absence information of pan-sequences. The presence of each pan-sequence (rows) was shown in red while the absence of each pan-sequence was shown in blue. [file Image_2.png]
