# Supplementary material for: Towards the Complete Goat Pan-Genome by Recovering Missing Genomic Segments From the Reference Genome
Source: Front Genet. 2019 Nov 15;10:1169. doi: 10.3389/fgene.2019.01169 (PMC6874019; doi:10.3389/fgene.2019.01169)
Supplement: Supplementary file 4 [file DataSheet_2.docx]

Supplementary Table S2. The statistics of *de novo* assemblies used for goat pan-genome construction.

|  | Species | Accession NO. | Contig N50 | Scaffold N50 | Net length |
| --- | --- | --- | --- | --- | --- |
| ARS1 | *Capra hircus* | GCA_001704415.1 | 26.2 Mbp | 87.2 Mbp | 2.92 Gb |
| CHIR2.0 | *Capra hircus* | GCA_000317765.2 | 73.5 kb | 9.09 Mb | 2.72 Gb |
| CSI1.0 | *Capra Siberica* | Not available | 76.9 kb | 15.1 Mb | 2.77 Gb |
| Argali1.0 | *Ovis ammon* | GCA_003121645.1 | 45.6 kb | 15.7 Mb | 2.66 Gb |
| Oori1 | *Ovis musimon* | GCF_000765115.1 | 39.7 kb | 2.2 Mb | 2.59 Gb |
| Oar4.0 | *Ovis aries* | GCA_000298735.2 | 150.4 kb | 100.0 Mb | 2.59 Gb |
| [CapAeg_1.0](https://www.ncbi.nlm.nih.gov/assembly/GCA_000978405.1) | *Capra aegagrus* | GCA_000978405.1 | 19.3 kb | 91.3 Mb | 2.59 Gb |
| [Caeg1](https://www.ncbi.nlm.nih.gov/assembly/GCA_000765075.1) | *Capra aegagrus* | GCA_000765075.1 | 51.8 kb | 1.7 Mb | 2.58 Gb |
| ALER1.0 | *Ammotragus lervia* | GCA_002201775.1 | 27.0 kb | 1.3 Mb | 2.64 Gb |
| ASM318257v1 | *Pseudois nayaur* | GCA_003182575.1 | 15.1 kb | 2.1 Mb | 2.63 Gb |
