# Supplementary material for: Towards the Complete Goat Pan-Genome by Recovering Missing Genomic Segments From the Reference Genome
Source: Front Genet. 2019 Nov 15;10:1169. doi: 10.3389/fgene.2019.01169 (PMC6874019; doi:10.3389/fgene.2019.01169)
Supplement: Supplementary file 5 [file DataSheet_3.docx]

Supplementary Table S3. Fossil calibration data used for the Caprini phylogenetic analysis.

| Calibrate node | Age (MYA) | Fossil taxon | reference |
| --- | --- | --- | --- |
| Crown Bovidae | 16-20 | Eotragus noyei | 1 |
| Caprini | 8.9-13.0 | Aragoral mudejar | 2 |
| Capra | >1.90 | Capra wodaramoya | 3 |
| Ovis | >2.42 | Sinocapra willdownsi | 4 |

1. Solounias N, Barry JC, Bernor RL, Lindsay EH, Raza SM (1995) The oldest bovid from the Siwaliks, Pakistan. Journal of Vertebrate Paleontology 15: 806-814

2. Van Dam JA, Alcala L, Alonso-Zarza AM, Calvo JP, Garces M, Krijgsman W (2001) The upper Miocene mammal record from the Teruel-Alfambra region (Spain). The MN system and continental stage/age concepts discussed. Journal of Vertebrate Paleontology 21: 367-385

3. Bibi F, Vrba E, Fack F (2012) A new african fossil caprin and a combined molecular and morphological bayesian phylogenetic analysis of caprini (Mammalia: Bovidae). Journal of Evolutionary Biology 25: 1843-1854

4. Mead JI, Taylor LH (2005) New species of Sinocapra (Bovidae, Caprinae) from the Lower Pliocene Panaca formation, Nevada, USA. Palaeontologia Electronica 8
